# Supplementary figures and images for: High-resolution biomechanical mapping of SMILE and SMILE with CXL using Brillouin microscopy: Insights into localized corneal stiffness preservation
Source: PLoS One. 2025 Dec 18;20(12):e0338302. doi: 10.1371/journal.pone.0338302 (PMC12714255; doi:10.1371/journal.pone.0338302)

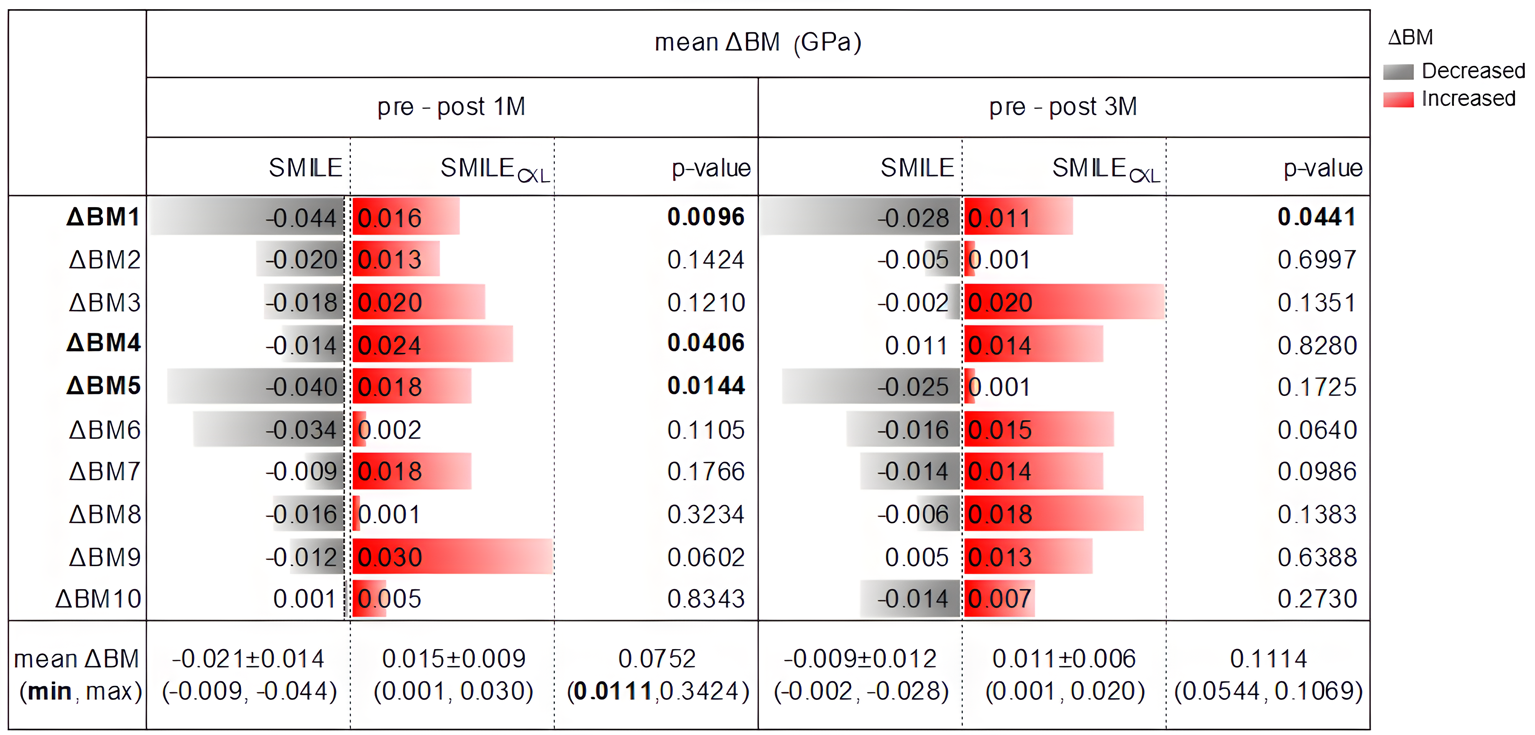

Supplement: S1 Fig — Color gradients indicate: gray for losses (ΔBM < 0, mainly SMILE), red for preservation/gains (ΔBM ≥ 0, mainly SMILECXL). Rows: BM1–BM10 and aggregates (mean/min/max); bolded p < 0.05. Highlights CXL’s protective effects (e.g., significant at BM1, BM5). Analyses: independent t-tests for inter-group comparisons (p < 0.05 exact). Data presented as mean (GPa). (TIF) [file pone.0338302.s001.tif]
