# Supplementary material for: High-resolution biomechanical mapping of SMILE and SMILE with CXL using Brillouin microscopy: Insights into localized corneal stiffness preservation
Source: PLoS One. 2025 Dec 18;20(12):e0338302. doi: 10.1371/journal.pone.0338302 (PMC12714255; doi:10.1371/journal.pone.0338302)
Supplement: Attachment — Submitted filename: Response to Reviewers.docx [file pone.0338302.s003.docx]

**Point-by-Point Response to Reviewers**

We sincerely thank the Editor and Reviewers for their insightful feedback and the decision for minor revision. We have addressed all comments with precise, evidence-based edits to enhance clarity and clinical relevance, without introducing unsubstantiated claims.

**Journal Requirements**

1. Please ensure that your manuscript meets PLOS ONE's style requirements, including those for file naming. The PLOS ONE style templates can be found at https://journals.plos.org/plosone/s/file?id=wjVg/PLOSOne_formatting_sample_main_body.pdf and <https://journals.plos.org/plosone/s/file?id=ba62/PLOSOne_formatting_sample_title_authors_affiliations.pdf>

**Response:** We have revised the manuscript to fully comply with PLOS ONE’s style requirements, including sentence case for the title, full affiliations without abbreviations, and corresponding author email format, as per the provided templates. No further issues were identified, but if overlooked, we appreciate specific guidance.

2. In the online submission form you indicate that your data is not available for proprietary reasons and have provided a contact point for accessing this data. Please note that your current contact point is a co-author on this manuscript. According to our Data Policy, the contact point must not be an author on the manuscript and must be an institutional contact, ideally not an individual. Please revise your data statement to a non-author institutional point of contact, such as a data access or ethics committee, and send this to us via return email. Please also include contact information for the third party organization, and please include the full citation of where the data can be found.

**Response:** As per the Data Policy, we have updated the Data Availability Statement to specify a non-author institutional contact: the Data Access Committee at Fatima Eye Clinic, Changwon, Republic of Korea (fatimaeye@naver.com). The full citation for data access is: "Data cannot be shared publicly due to legal restrictions under South Korean law, including the Personal Information Protection Act, and hospital policies at Fatima Eye Clinic, which prohibit the external sharing of clinical patient data, even when anonymized, without additional ethical review. Data are available upon reasonable request from the Fatima Eye Clinic Data Access Committee (fatimaeye@naver.com), subject to institutional approval for researchers who meet the criteria for access to confidential data." This revision has been added in lines 467-474 and emailed to plosone@plos.org as instructed.

3. If the reviewer comments include a recommendation to cite specific previously published works, please review and evaluate these publications to determine whether they are relevant and should be cited. There is no requirement to cite these works unless the editor has indicated otherwise.

**Response:** No specific publications were recommended by the reviewers for citation. We have ensured the reference list is complete and accurate without additional changes.

4. Please review your reference list to ensure that it is complete and correct. If you have cited papers that have been retracted, please include the rationale for doing so in the manuscript text, or remove these references and replace them with relevant current references. Any changes to the reference list should be mentioned in the rebuttal letter that accompanies your revised manuscript. If you need to cite a retracted article, indicate the article’s retracted status in the References list and also include a citation and full reference for the retraction notice.

**Response:** The reference list has been reviewed for completeness and accuracy. No retracted articles are cited. Minor additions (e.g., Randleman et al., 2008 [3]; Wollensak et al., 2009 [8]; Hersh et al., 2017 [11]; Randleman et al., 2017 [28]) were made to address reviewer comments on clinical translation, increasing the total from 26 to 29 while ensuring 100% accuracy and relevance based on PubMed verification.

5. **Additional Editor Comments:** Please address the minor comments from the Reviewers fully with edits to the manuscript.

**Response:** We have addressed all minor comments from the reviewers with corresponding edits to the manuscript, as detailed below.

**Reviewer # 1**

The study addresses an important clinical issue. The use of BOSS for high-resolution mapping is novel and fills a gap compared with prior LASIK-focused or aggregate-metric studies.

1. The introduction should more clearly highlight the clinical implications of localized biomechanical measurements and provide stronger justification for the accelerated epi-on CXL protocol.

**Response:** We have revised the Introduction (lines 100–107) to more explicitly highlight the clinical implications of localized biomechanical measurements, such as identifying regional stiffness variations that may inform ectasia risk assessment in high-myopia patients, and provided stronger justification for the accelerated epi-on CXL protocol, citing relevant literature (e.g., Wollensak et al., 2009 [8]).

2. After propensity score matching, only 17 eyes per group remained, which markedly reduces statistical power and raises the risk of overfitting. This limitation should be explicitly acknowledged. In addition, the authors should clarify how bilateral eyes, if included, were handled to avoid violating independence assumptions.

**Response:** We have explicitly acknowledged the limitations of the small post-PSM sample size (17 eyes/group) in the Limitations section (lines 433–442), including reduced power and overfitting risk. To address the violation of independence in the between-group comparison of pre- and postoperative ∆BM due to the inclusion of bilateral eyes from some participants, generalized estimating equations (GEE) with an exchangeable correlation structure were used to account for within-person correlation in clustered data from bilateral eyes. We clarified this in the Abstract (lines 27–31) and the main text (lines 231–235).

3. Some of the language in the results and discussion is too strong given the modest magnitude of biomechanical changes. The conclusions should be tempered accordingly.

**Response:** We have tempered the language in the Abstract (lines 53–59) and the main text (lines 450–456) to reflect the modest magnitude of changes, avoiding overstatements while maintaining data-supported conclusions. Additionally, to ensure consistency, we revised the introduction’s final paragraph (lines 113-120) by softening references to personalized treatments and ectasia risk, aligning with the study’s focus on observed biomechanical dynamics without implying direct clinical applications not investigated.

4. The focus on ectasia risk mitigation is valuable, but the authors should clarify how the small changes observed in Brillouin modulus may translate into clinically meaningful outcomes such as ectasia prevention or long-term refractive stability.

**Response:** We have clarified in the Discussion (lines 433–438) how the observed small BM changes may translate to clinical outcomes, such as ectasia prevention and refractive stability, by referencing studies showing that even modest stiffness increases correlate with reduced ectasia incidence in long-term follow-up (e.g., Hersh et al., 2017 [11]; Randleman et al., 2017 [28]). Our observed shift (0.01-0.04 GPa) align with similar scales in these studies (e.g., 0.017-0.035 GPa resections post-LASIK correlating with biomechanical stability), suggesting that such small BM variations may indicate early changes relevant for monitoring refractive outcomes. This underscores the research value in precision mapping for early intervention, enhancing clinical translation without overstatement. We also tempered related language in the Introduction (lines 113-120) to avoid overstating ectasia risk mitigation, focusing instead on biomechanical stability insights.

**Reviewer # 2**

I read with great interest the manuscript regarding investigating corneal biomechanical changes during SMILE/SMILE+CXL using Brillouin microscopy. The study’s main strengths include the application of Brillouin microscopy to evaluate corneal biomechanics after SMILE and its combination with CXL, as well as the temporal analysis of biomechanical changes over time. Please find my comments below.

1. Lines 54–56: This conclusion appears too strong and not in line with the results. The study demonstrates the potential effect of combining SMILE with CXL on corneal biomechanical stiffness as measured by BOSS. However, the utility of BOSS for personalized CXL planning or identification of at-risk corneal regions while alluded to, have not been directly investigated in this work.

**Response:** We have revised the conclusions in lines 53–59 (Abstract) and lines 450–456 (main text) to align with the results, emphasizing the potential rather than direct investigation of BOSS for personalized CXL planning. To maintain consistency across the manuscript, we similarly adjusted the Introduction’s final paragraph (lines 113-120) by removing allusions to personalized paradigms and ectasia risk attenuation, ensuring claims remain grounded in the study’s findings.

2. Lines 23, 27, 116, 128, and 137: While it is commendable that propensity score matching was performed on an initial cohort of 358 eyes, resulting in two matched groups of 17 eyes each, the final sample size is mentioned much later and in a relatively understated manner in both the abstract and methods. I recommend revising these sections so that the final post-matching sample size is stated immediately after the initial cohort size to improve clarity, especially for readers skimming the paper.

**Response:** We have revised the Abstract (lines 22–27) and the main text (lines 136–138) to state the final post-matching sample size (17 eyes each) immediately after the initial cohort size for improved clarity.

3. Please define BOSS at its first mention and include the manufacturer and model information.

**Response:** We have defined BOSS at its first mention in lines 93–94 (Introduction) and included manufacturer information (Intelon Optics, Woburn, MA, USA) in line 188 (Materials & Methods).
